# Sol–Gel Technology Applied to Materials Science: Synthesis, Characterization and Applications

**DOI:** 10.3390/ma17020462

**Published:** 2024-01-18

**Authors:** Aleksej Zarkov

**Affiliations:** Institute of Chemistry, Vilnius University, Naugarduko 24, LT-03225 Vilnius, Lithuania; aleksej.zarkov@chf.vu.lt

The rapid advances in technologies around the globe necessitate the development of new materials, nanostructures, and multicomponent composites with specific chemical and physical properties that can meet the requirements of modern technologies. Using appropriate synthetic approaches is crucial for the preparation of inorganic materials with designed microstructure and properties. Among the different technologies currently available, the sol–gel method is very well known for its versatility, simplicity, and time- and cost-efficiency. The mixing of starting materials on an atomic level provides high homogeneity and stoichiometry of the products, facilitating the fabrication of high-quality materials at low temperatures. The versatility of the Sol–Gel method allows for the development of materials for a wide range of applications in electronics, optoelectronics, catalysis, biomedicine, and many other areas. The scope of this Special Issue of Materials, entitled “Sol–Gel Technology Applied to Materials Science: Synthesis, Characterization and Applications”, is focused on, but not limited to, the preparation, characterization, and application of functional inorganic materials, as well as hybrid materials, which are important in the field of catalysis, electronics, optics, biomedicine, etc.

Due to the uniqueness of the sol–gel approach, it can be used for the preparation of both powders and thin films. Several contributions in this Special Issue investigate powdered sol–gel-derived materials. Ghezali et al. [1] investigated compositionally complex BaMnO_3_-based perovskite-type materials. Two series of materials with the chemical formulas Ba_0.9_A_0.1_MnO_3_ (BM-A) and Ba_0.9_A_0.1_Mn_0.7_Cu_0.3_O_3_ (BMC-A) (A = Mg, Ca, Sr, Ce, and La) were synthesized and applied for soot oxidation in simulated gasoline direct injection engine exhaust conditions. The soot conversion data reveal that Ba_0.9_La_0.1_Mn_0.7_Cu_0.3_O_3_ (BMC-La) is the most active catalyst in an inert (100% He) reaction atmosphere, and Ba_0.9_Ce_0.1_MnO_3_ (BM-Ce) is the best catalyst if a low amount of O_2_ (1% O_2_ in He) is present.

Karnaukhov et al. [2] used the sol–gel technique to prepare the two-component oxide system Cu–Mg–O, where MgO acts as the oxide matrix, and CuO is an active chemical looping component. The reduction behavior of the Cu–Mg–O system was examined in nine consecutive reduction/oxidation cycles. The main characteristics of the oxide system underwent noticeable changes during the first reduction/oxidation cycle. Starting from the third cycle, the system stabilized, providing the uptake of similar hydrogen amounts within the same temperature range.

The design, synthesis, and tuning of the properties of magnetic materials are among the important tasks of materials science [3]. Karoblis et al. [4] investigated the influence of processing conditions on the phase purity and the structural and magnetic properties of sol–gel-derived yttrium-doped MgFe_2_O_4_. The authors found that the iron content in the initial reaction mixture significantly contributed to the phase purity of the final products substituted with aliovalent Y^3+^ ions. According to the Mössbauer spectroscopy studies, with the increase in the dopant amount in solid solutions, the amount of iron in the tetrahedral position increased. The coercivity as well as saturation magnetization decreased with the increase in the yttrium content.

Mejia-Estrella et al. [5] aimed to obtain a viable photocatalytic material able to oxidize organic pollutants under the visible light spectrum. For this purpose, the authors fabricated different Pt-TiO_2_ and Pt-TiO_2_-CeO_2_ composite materials employing sol–gel and impregnation methods. The obtained materials were tested for their photocatalytic oxidation activity on a herbicide 2,4-dichlorophenoxyacetic acid, frequently used in agriculture. The activity of the materials reached a removal efficiency of 98% of the initial concentration of the pollutant in 6 h.

Monros et al. [6] applied the sol–gel technique for the preparation of Ca- and Cr-doped BiVO_4_-based compounds. This multifunctional host material has raised considerable interest in the scientific community as a wideband semiconductor with photocatalytic activity, a material with high NIR reflectance for camouflage and cool pigments, and a photoanode for photoelectrochemical seawater splitting. Ca- or Cr-doped BiVO_4_ pigments have been prepared for paints and glazes, with colors ranging from turquoise to black depending on the processing route.

The deposition of thin films employing the sol–gel approach allows for the fabrication of coatings on both flat and shaped surfaces. Covei et al. [7] studied the influence of the deposition parameters on the properties of TiO_2_ thin films on spherical substrates. The complex influence of the substrate morphology, the sol dilution with ethanol, and the number of layers on the structure, morphology, chemical composition, and photocatalytic performance of TiO_2_ thin films were investigated. As a result, photocatalytically active TiO_2_ thin films with high surface area were deposited on spherical beads (2 mm diameter), ensuring easy recovery from wastewater.

Wojtasik et al. [8] fabricated photocatalytically active ZnO coatings on soda lime glass substrates using the sol–gel method and the dip-coating technique. Their study aimed to determine the effect of the duration of the sol aging process on the properties of the fabricated ZnO films. The photocatalytic properties of ZnO layers were studied by observing the degradation of methylene blue dye in an aqueous solution under UV illumination. The strongest photocatalytic activity was observed for the coatings produced from a sol system that was aged over 30 days. These layers also had the highest porosity (37.1%) and the largest water contact angle (68.53°).

Crespo-Monteiro et al. [9] investigated a sol–gel procedure that allows for the direct micro–nanostructuring of ZrO_2_ layers without physical or chemical etching processes, using optical or nanoimprint lithography. The synthesis of ZrO_2_ and the micro–nanostructuring process were presented by masking, colloidal lithography, and nanoimprint lithography on glass and plastic substrates as well as on plane and curved substrates.

The sol–gel approach also allows for the easy modification and functionalization of the final products. Toirac et al. [10] investigated biodegradable sol–gel coatings as a promising method for the controlled release of antibiotics for the local prevention of infection in joint prostheses. Sols were prepared from a mixture of MAPTMS and TMOS silanes, tris(tri-methylsilyl)phosphite, and eight different individually loaded antimicrobials. The results revealed that the coatings had a microscale roughness attributed to the accumulation of antibiotics and organophosphites in the surface protrusions; no evidence was found for the existence of chemical bonds between antibiotics and the siloxane network.

Although the sol–gel method is mostly used for the synthesis of oxide materials, the synthesis of halides is also possible. Pellegrino et al. [11] successfully applied the sol–gel process for the fabrication of Eu-doped CaF_2_ thin films as down-shifting layers for solar cell applications. The Ca(hfa)_2_∙diglyme∙H_2_O and Eu(hfa)_3_∙diglyme were used as precursors and mixed in various proportions in a water–ethanol solution. The optimization of deposition process parameters in terms of annealing temperature, substrate nature, and doping ion percentage was performed. The down-shifting properties were validated by taking luminescence measurements under UV excitation.

## References

[1] Ghezali N., Díaz Verde Á., Illán Gómez M.J. (2023). Screening Ba_0.9_A_0.1_MnO_3_ and Ba_0.9_A_0.1_Mn_0.7_Cu_0.3_O_3_ (A = Mg, Ca, Sr, Ce, La) Sol-Gel Synthesised Perovskites as GPF Catalysts. Materials.

[2] Karnaukhov T.M., Veselov G.B., Cherepanova S.V., Vedyagin A.A. (2022). Sol-Gel Synthesis and Characterization of the Cu-Mg-O System for Chemical Looping Application. Materials.

[3] Karoblis D., Stewart O.C., Glaser P., El Jamal S.E., Kizalaite A., Murauskas T., Zarkov A., Kareiva A., Stoll S.L. (2023). Low-Temperature Synthesis of Magnetic Pyrochlores (R_2_Mn_2_O_7_, R = Y, Ho–Lu) at Ambient Pressure and Potential for High-Entropy Oxide Synthesis. Inorg. Chem..

[4] Karoblis D., Mazeika K., Raudonis R., Zarkov A., Kareiva A. (2022). Sol-Gel Synthesis and Characterization of Yttrium-Doped MgFe_2_O_4_ Spinel. Materials.

[5] Mejia-Estrella I.A., Pérez Larios A., Sulbarán-Rangel B., Guzmán González C.A. (2022). Reaching Visible Light Photocatalysts with Pt Nanoparticles Supported in TiO_2_-CeO_2_. Materials.

[6] Monrós G., Llusar M., Badenes J.A. (2023). High NIR Reflectance and Photocatalytic Ceramic Pigments Based on M-Doped Clinobisvanite BiVO_4_ (M = Ca, Cr) from Gels. Materials.

[7] Covei M., Bogatu C., Gheorghita S., Duta A., Stroescu H., Nicolescu M., Calderon-Moreno J.M., Atkinson I., Bratan V., Gartner M. (2023). Influence of the Deposition Parameters on the Properties of TiO_2_ Thin Films on Spherical Substrates. Materials.

[8] Wojtasik K., Zięba M., Tyszkiewicz C., Pakieła W., Żak G., Jeremiasz O., Gondek E., Drabczyk K., Karasiński P. (2023). Zinc Oxide Films Fabricated via Sol-Gel Method and Dip-Coating Technique—Effect of Sol Aging on Optical Properties, Morphology and Photocatalytic Activity. Materials.

[9] Crespo-Monteiro N., Valour A., Vallejo-Otero V., Traynar M., Reynaud S., Gamet E., Jourlin Y. (2022). Versatile Zirconium Oxide (ZrO_2_) Sol-Gel Development for the Micro-Structuring of Various Substrates (Nature and Shape) by Optical and Nano-Imprint Lithography. Materials.

[10] Toirac B., Garcia-Casas A., Monclús M.A., Aguilera-Correa J.J., Esteban J., Jiménez-Morales A. (2022). Influence of Addition of Antibiotics on Chemical and Surface Properties of Sol-Gel Coatings. Materials.

[11] Pellegrino A.L., Milan E., Speghini A., Malandrino G. (2023). Fabrication of Europium-Doped CaF_2_ Films via Sol-Gel Synthesis as Down-Shifting Layers for Solar Cell Applications. Materials.

